# Youth and Young Adults’ Perspectives on Augmented Reality–Driven Vaping Cessation Interventions: Interpretive Description Study

**DOI:** 10.2196/79674

**Published:** 2025-12-23

**Authors:** Karlee Fonteyne, Elizabeth Keys, Mohammad Khalad Hasan, Laura Struik

**Affiliations:** 1 School of Nursing, University of British Columbia, 1147 Research Road, Kelowna, BC, V1V 1V7, Canada, 1 250-807-9972; 2 Department of Computer Science, Mathematics, Physics and Statistics, University of British Columbia, Kelowna, BC, Canada

**Keywords:** vaping cessation, cessation support, augmented reality, AR, nicotine, nicotine dependency, youth, young adults, e-cigarettes, mHealth, apps

## Abstract

**Background:**

Vaping among youth and young adults has become a significant public health issue, with increasing prevalence and associated health risks. Despite awareness of these risks, many youth and young adults struggle to quit due to complex social pressures, stress, and a lack of tailored interventions. Digital tools, including augmented reality (AR), offer an opportunity to address these challenges by creating engaging and personalized support systems.

**Objective:**

The aim of this study was to determine what can be learned from youth and young adult vapers who are motivated to quit vaping to inform the design of mobile app–based AR intervention strategies.

**Methods:**

This qualitative study applied an interpretive description (ID) approach to explore youth and young adults’ perspectives on vaping cessation and their preferences for digital intervention features. Semistructured interviews were conducted with participants (N=12) who shared their experiences with vaping, quitting attempts, and ideas for app-based AR support. Reflexive thematic analysis and ID were used to code the data and identify patterns, resulting in the generation of themes that reflected the individualized and contextual nature of vaping cessation.

**Results:**

The findings collectively yielded four major themes: (1) social and cultural context play a role in youth and young adults’ experiences of cessation, (2) quitting vaping is an individual endeavor that does not always mean success, (3) digital support as a bridge between individual and social needs, and (4) AR as a catalyst for personalized support. These themes address the motivations, challenges, and opportunities identified by participants in their cessation journeys, as well as their perspectives on integrating AR technology as a supportive tool. Our findings reveal that vaping cessation is a deeply personal process influenced by internal motivations (eg, health improvement and personal milestones) and external factors (eg, social context). Participants identified AR as a promising app-based tool for cessation support, with interest in potential AR-integrated features such as gamified rewards, health visualizations, and anonymous support. Youth and young adults emphasized the need for sensitive design to avoid negative or punitive content.

**Conclusions:**

This study provides actionable insights for designing youth and young adult–centered digital health tools that leverage app-based AR to support vaping cessation. By addressing the unique sociocultural and behavioral needs of youth and young adults, app-based AR interventions can bridge gaps in traditional cessation strategies. These findings contribute to the development of innovative public health approaches aimed at reducing vaping prevalence in vulnerable populations.

## Introduction

### Background

Vaping, particularly among youth and young adults, represents a growing epidemic with concerning health and societal implications. In Canada, the prevalence of vaping has risen sharply in the last decade, with 30% of adolescents aged 15‐19 years and 48% of young adults aged 20‐24 years reporting having ever tried vaping, and 13% and 18%, respectively, reporting vaping within the past 30 days [1]. These rates reflect a nearly 120% increase compared to prelegalization figures in 2017, underscoring the urgency of effective cessation interventions [2].

Initially marketed as a harm-reduction tool for adult smokers, vaping has gained traction among nonsmoking youth and young adults due to its perceived safety, appealing flavors, and sleek, portable devices [3-6]. Social media, peer influences, and cultural norms further exacerbate the problem by glamorizing vaping as a socially acceptable behavior [6-8]. Emerging evidence highlights significant health risks associated with vaping, including nicotine dependence and respiratory and cardiovascular complications [9,10]. At the same time, Youth and young adults are navigating a broader mental health crisis, and many turn to vaping as a coping mechanism for stress and anxiety despite emerging evidence linking vaping to worsening psychological distress [11,12].

Despite increasing motivation to quit [13,14], many youth and young adults face unique barriers to cessation, including nicotine dependence, social reinforcement, and a lack of appealing support tools [5,15,16]. Traditional cessation methods, which are often adapted from adult-focused smoking programs, tend to be rigid, abstinence-oriented, and disconnected from the lived realities of youth and young adults. These approaches frequently neglect the digital and social contexts that shape youth behavior and fail to resonate with their preferences for nonjudgmental, flexible, and emotionally supportive tools [5,17,18]. Given this mismatch, there is a pressing need for novel, youth-driven solutions that prioritize harm reduction, self-compassion, and gradual behavior change.

Augmented reality (AR) represents a novel opportunity to meet these preferences. AR technology overlays digital elements onto real-world environments through smartphones or tablets, creating immersive and interactive experiences that can disrupt habitual behaviors and support real-time decision-making [18-20]. Parekh et al [21] present a systematic review and meta-analysis of AR applications in the sectors of entertainment or gaming, medicine, and retail, highlighting their widespread potential. Several popular mobile-based apps, including Pokémon Go, Snapchat, Google Maps, and Google Translate, use AR technology; however, many users may not be aware of this fact or the underlying principles of the technology. Vinci et al [18] argue that AR does not share some of the restrictions of other immersive technologies like virtual reality and can be more cost- and time-effective in development because only an object is created, not a whole scene; this may also lend to a higher degree of realism for the object.

Despite AR’s potential, it remains largely understudied and underapplied in contemporary health interventions. Much of the research to date focuses on the application of AR to support smoking cessation. AR has demonstrated high usability, acceptability, and preliminary effectiveness in an app-based smoking cessation intervention using cue exposure therapy (CET) to disrupt associations between environmental triggers and cravings [20]. Building on these findings, Poudel et al [22] outlined a randomized controlled trial to evaluate an AR-CET app intervention at scale. Similar cue-reactive mechanisms have been documented in the vaping literature. Experimental studies demonstrate that exposure to vaping cues increases craving, attentional bias, and activation in reward-related neural circuits among young adults [23-25], providing evidence that vaping behavior is strongly cue-driven in ways analogous to smoking. Emerging evidence also suggests that repeated exposure to vaping cues may attenuate craving over time, supporting the theoretical relevance of CET-informed approaches for vaping cessation. Beyond CET, Wang and Yao [26] successfully used AR to increase the risk perception of vaping by enhancing introspective awareness and reducing temporal and hypothetical distances associated with the behavior. AR features may be especially valuable for youth and young adults, who are highly engaged with mobile technology and often prefer dynamic, real-world interventions over static educational resources [4,5,17,27,28].

To date, no research has explored the potential of app-based AR for vaping cessation, particularly among youth and young adults [29,30]. This study addresses this critical gap by investigating youth and young adults’ perspectives, preferences, and experiences with vaping cessation to inform the development of AR app-based mobile interventions. By centring on the voices of youth and young adults, this research aims to advance the design of innovative, practical, and youth-driven digital tools to support vaping cessation.

### Research Aim

This study aimed to explore the experiences and perspectives of youth and young adults who are motivated to quit vaping, with the goal of informing the development of AR-driven mobile interventions. By focusing on this demographic’s unique needs, motivations, and challenges, the research aimed to generate practical insights that can guide the design of innovative and engaging cessation tools. The overarching goal was to identify key features and design considerations that would optimize the effectiveness and relevance of AR app-based interventions for youth and young adults.

## Methods

### Interpretive Description

This study used interpretive description (ID), a qualitative methodology designed to generate clinically relevant insights for applied health research [31]. ID is grounded in constructivist epistemology, which acknowledges that knowledge is co-constructed through interactions between researchers and participants. Unlike other qualitative methodologies that adhere to rigid theoretical frameworks, ID emphasizes flexibility, allowing researchers to explore both shared and individual experiences to develop actionable findings [31,32]. ID was particularly suited to this study’s focus on vaping cessation among youth and young adults, as it enabled a nuanced understanding of their experiences and preferences within broader social and technological contexts.

### Ethical Considerations

This study was granted ethics approval by the University of British Columbia Okanagan Behavioral Research Ethics Board (#H24-00545) in the spring of 2024. The recruitment process and all interview sessions occurred concurrently in the summer and fall of 2024. Informed consent was obtained from all participants, which was voluntary and revisited throughout the research process. Participants were provided with information pertaining to the study and a consent form embedded within the Qualtrics intake questionnaire. The consent form emphasized voluntary participation, the purpose of the study, potential risks, and the right to withdraw at any point without consequences. Participants were also informed about the potential emotional impact of discussing their vaping experiences, and support resources were shared. Youth and young adults were provided with an email to contact a researcher in case of additional questions or to receive more information about the study.

### Participants and Procedure

Participants were purposively recruited to ensure alignment with the study’s aims. Inclusion criteria were as follows: (1) aged 16‐24 years, (2) currently vaping, (3) motivated to quit vaping, (4) proficient in English, and (5) residing in Canada. The inclusion criteria for this study ensured the relevance and feasibility of the research. Participants aged 16 to 24 years were selected because this age range represents a critical developmental period where vaping behaviors are often initiated, and cessation efforts are particularly impactful [16]. Additionally, this demographic represents Canada’s largest population of vape users [1]. Requiring participants to be current vapers motivated to quit aligned with the study’s focus on understanding vaping cessation. Motivation to quit vaping was assessed via a “yes or no” question on the intake screener.

Recruitment was conducted via professional networks, outreach to teachers and public health professionals, and email invitations to past research participants. Recruitment materials, including digital poster advertisements, were shared on social media platforms and via email lists. Many participants were recruited from a list of previous research participants in LS’s laboratory who agreed to be contacted for future research. Past participants were contacted directly via the email address they had previously provided. Interested participants completed a Qualtrics survey to screen for eligibility and collect data on demographic characteristics, vaping history and behavior, dependence, motivation to quit vaping, app use, and familiarity with immersive technologies. Combining these items into a single Qualtrics survey (Multimedia Appendix 1) helped to streamline the process and prevent participant attrition. Researchers emailed interested participants who successfully completed the Qualtrics to schedule a Zoom (Zoom Communications, Inc) interview. All interviewed participants were offered honoraria (CAD $50 [US $36.31] e-gift card) for their time and contributions.

For ID, there is no preset quota for an appropriate sample size, as the focus is on generating rich, applicable insights rather than achieving statistical representation [31]. Rather than relying on data saturation, which assumes a finite set of themes that will eventually be exhausted, this study adopted the principle of information power [33]. Information power suggests that the more relevant and rich the data, the fewer participants are required, with sample size influenced by study aim, sample specificity, theoretical application, quality of dialogue, and analytical approach. The focused nature of this study, combined with a highly specific sample selected based on clear inclusion criteria, supported the feasibility of a smaller yet information-rich sample. Additionally, a manageable sample size enabled in-depth, inductive analysis, ensuring that the findings were meaningful rather than overly broad. Given the understudied nature of vaping cessation among youth and young adults, an initial estimate of 15‐20 participants was made.

### Data Collection and Analysis

Data were collected through semistructured interviews conducted via Zoom, ensuring flexibility and accessibility for geographically dispersed participants across Canada. Semistructured interviews were conducted using a carefully designed interview guide developed in collaboration with an interdisciplinary team and a youth co-researcher (Multimedia Appendix 2). The guide explored topics like participants’ vaping behaviors, motivations for quitting, experiences with existing cessation resources, and perspectives on the integration of AR technology into vaping cessation interventions. To facilitate meaningful engagement and ensure informed discussions, participants were introduced to AR technology before the interviews. Recognizing that many individuals are unfamiliar with AR or confuse it with other immersive technologies, such as virtual reality, participants were sent a brief email explaining the technology. Participants were also encouraged to interact with a free AR app, such as Pokémon Go, prior to their interview session to provide a point of reference for the technology’s capabilities, limitations, and potential applications.

The whiteboard function within the Zoom platform was leveraged during the semistructured interviews to further engage participants in the co-design process. Participants were shown the virtual whiteboard (Multimedia Appendix 3), which displayed four potential AR-based features, accompanied by brief explanations of each concept and supporting images. The proposed features were determined in collaboration with an interdisciplinary team member and computer scientist MKH. The goal of the whiteboard activity was to add clarity to the ideas and increase the interactivity of the interview [34]. To that point, Dewitz [34] highlights the potential of visual participatory approaches in enhancing interview settings and facilitating the gathering of rich data. Participants were asked to rank the four features (1=strongest idea to 4=weakest idea), and a screenshot of their choices was retained at the end of the interview.

All interviews were conducted by KF, each lasting approximately 45‐60 minutes. With participants’ informed consent, interviews were audio-recorded to ensure accurate and comprehensive capture of participants’ narratives. All data were deidentified to protect confidentiality, and audio recordings were transcribed verbatim using Otter.ai. Transcripts were manually reviewed and corrected by KF to ensure accuracy and to achieve a high degree of familiarity with the data [31,35,36].

In ID, data are viewed as constructed rather than collected, highlighting the interpretive paradigm by reflecting the researcher’s active role in choosing what counts as data and is subsequently analyzed as such [31,36]. Data were collected and analyzed concurrently to enrich data collection and analysis through this iterative process. After 10 interviews, no substantively new insights were emerging in relation to the research question. To ensure analytical rigor and confirm sufficiency, 2 additional interviews were conducted, reinforcing the determination that sufficient information power had been achieved. At this stage, further data collection was unlikely to yield significantly new perspectives while still maintaining feasibility within the study scope. By aligning with the principles of ID, information power, and reflexive thematic analysis (RTA), this approach ensured that the selected sample size struck a balance among data richness, methodological rigor, and practical constraints while allowing for a deep and meaningful exploration.

The data were analyzed using RTA, a qualitative method that aligns with ID by facilitating the exploration of complex and nuanced participant experiences [31,35,36]. RTA’s flexibility and iterative nature allowed themes to emerge organically, capturing the depth of participants’ narratives while addressing the study’s objectives. Braun and Clarke’s [35] six-phase framework guided the analysis process. First, familiarization with the data involved transcription and repeated readings of focus group and interview transcripts, during which initial impressions were documented in a reflexive journal. Coding was conducted manually to enable a deeper engagement with the data, while NVivo 14 software (Lumivero) was used to organize and manage codes. The coding process focused on identifying meaningful patterns and relationships relevant to the research questions. Initial codes were grouped into candidate themes, which were iteratively refined through reviews of transcripts and reflexive memos. Discussions between LS and KF further supported the refinement process, ensuring that the themes reflected participant perspectives and broader social and cultural contexts. Themes were then defined and named to capture their essence, integrating participant language where possible to maintain authenticity. The final themes were presented as a coherent narrative supported by illustrative participant quotes. The analytical approach emphasized rigor, reflexivity, and alignment with the principles of ID, ensuring that the findings offer actionable insights for designing youth and young adult–driven AR interventions for vaping cessation.

### Gender-Based Analysis

Given documented gender-based differences in vaping behaviors [27], this study included an analysis disaggregated by gender to explore potential trends, such as variations in motivations for cessation or preferences for AR features. Gender was self-reported during demographic data collection, with all females identifying as women and all males identifying as men. Reflexive memos documented gender-related observations, which were revisited during theme refinement to ensure gendered patterns were considered.

### Quality and Rigour

To ensure methodological rigor, the study adhered to Thorne’s [31] quality criteria for ID research. Epistemological integrity was maintained by aligning the constructivist foundation with the study design and analysis. Representative credibility was achieved through purposive sampling and rich, thick descriptions of participant narratives, as well as triangulation between the interview transcripts, whiteboard interactions, and questionnaire responses. Analytic logic was demonstrated through an audit trail of decisions made during data collection and analysis. Finally, reflexivity was prioritized throughout the research process, with KF journaling reflections and acknowledging their positionality as a nurse researcher with a professional interest in vaping cessation.

## Results

### Description of the Sample

The findings (see Table 1) provide a detailed profile of the participants’ vaping histories, cessation strategies, and primary motivations, offering valuable insights into the diversity of experiences within the study cohort. A total of 19 individuals completed the Qualtrics survey; however, 7 participants were lost to attrition due to either not responding to emails to schedule an interview or failing to attend their scheduled session. Participants (N=12, mean age 21.4 years) included a mix of self-identified genders (7 men, 5 women). The small sample size limited the capacity for a robust gender-based analysis, and no distinct trends were identified. This does not imply that gender differences are absent, but highlights the need for future research with larger, more diverse samples to investigate these nuances systematically. Participants indicated varying durations of vaping, ranging from 1‐3 years to over 3 years. The frequency of vaping also varied significantly, from occasional use (once per week or less) to several times per day. Cessation attempts were common, with most participants reporting 1‐4 attempts, although a few indicated more frequent efforts (5‐10 attempts). The strategies used were similarly varied, encompassing gradual reduction, cold turkey, cessation apps, nicotine replacement therapy, and professional counseling. Primary motivations for cessation spanned personal health concerns, financial pressures, social influences, and shifts in perceptions about vaping’s appeal. The majority of participants indicated that they were somewhat familiar with AR technology and had some knowledge of its applications.

**Table 1.** Participant characteristics and vaping history (N=12).

ID	Gender	Age (years)	Time vaping (years)	Frequency of vaping	Cessation attempts	Cessation strategies	Primary motivation	Familiar with AR^a^ (no/somewhat/yes)
1	M	19	>3	Several times per day	1‐4 attempts	Gradual reduction, cold turkey, and NRT^b^	Financial	No
2	M	21	1‐3	About once per day	—^c^	—	Health	Yes
3	W	24	1‐3	Several times per day	1‐4 attempts	Gradual reduction, cessation apps, and NRT	Health	Yes
4	W	23	>3	Several times per day	1‐4 attempts	Cold turkey, cessation apps, and NRT	Social pressures	Somewhat
5	M	22	>3	Once per week or less	1‐4 attempts	Gradual reduction, NRT, and professional counseling	Health	No
6	W	21	>3	Several times per day	1‐4 attempts	Gradual reduction, cold turkey, cessation apps, and NRT	Health	Somewhat
7	M	22	>3	A few times per week	5‐10 attempts	Gradual reduction, cold turkey, and tolerance breaks	Health	Somewhat
8	M	21	1‐3	Several times per day	1‐4 attempts	Gradual reduction	Health	Somewhat
9	W	21	1‐3	Several times per day	1‐4 attempts	Gradual reduction and tolerance breaks	Health	Somewhat
10	M	18	1‐3	Several times per day	1‐4 attempts	Gradual reduction and tolerance breaks	Shift in perception	Somewhat
11	M	21	1‐3	About once per day	1‐4 attempts	Gradual reduction, tolerance breaks, and cessation apps	Shift in perception	Somewhat
12	W	24	>3	Several times per day	5‐10 attempts	Cold turkey, cessation apps, and NRT	Financial	Somewhat

a AR: augmented reality.

b NRT: nicotine replacement therapy.

c Not applicable.

### Qualitative Themes

The findings collectively yielded four major themes: (1) social and cultural context play a role in youth and young adults’ experiences of cessation, (2) quitting vaping is an individual endeavor that does not always mean success, (3) digital support as a bridge between individual and social needs, and (4) AR as a catalyst for personalized support. These themes address the motivations, challenges, and opportunities identified by participants in their cessation journeys, as well as their perspectives on integrating AR technology as a supportive tool. To illustrate these themes, participant quotes support the results, which provide a nuanced understanding of their lived experiences and preferences. By grounding the findings in participants’ voices, this section highlights critical insights to inform the design of engaging, practical, and youth and young adult–driven AR interventions for vaping cessation.

#### Social and Cultural Context Play a Role in Youth and Young Adults’ Experiences of Cessation

Participants described vaping as a normalized and pervasive behavior within their social environments, shaping both their initiation into the habit and their cessation attempts. Most youth and young adults indicated that they started vaping in social settings due to exposure, often viewing it as an expected part of social interactions. Vaping was frequently portrayed as an accepted and routine activity in many environments, creating a sense of normalcy that reinforced its persistence. When asked about their quitting journeys, many participants highlighted how their social environment and peers acted as barriers to cessation.

*It’s almost like there’s like, a push not to quit [...] it’s not that they’re like, constantly telling me you shouldn’t quit, but it’s like, they’ll offer me vapes constantly or something like that. So, it makes it harder, it’s still doable, like you can always just say no, but it does make it much harder.*

[#5, man, 22 years]

The normalization of vaping has created significant barriers to quitting, as participants often felt that ceasing to vape could isolate them socially or disrupt their sense of belonging. One participant expressed concerns about being excluded by colleagues at work, highlighting how social contexts can reinforce vaping behaviors and make quitting more difficult.

*I feel like vaping is just a very normalized thing amongst young people [...] in my work, there’s actually like a designated smoke space for people outside, they would go to smoke, and they would go to vape [...] I feel like stopping would make me kind of like a social outcast.*

[#1, man, 19 years]

In many cases, participants deliberately distanced themselves from environments, social settings, or relationships that normalized or encouraged vaping. For some, this involved avoiding social gatherings or workspaces where vaping was prevalent, while for others, it meant disengaging from friendships with peers who vaped regularly. Although these measures were often helpful, they came with personal sacrifices: “My peer group has been negative. They haven’t been helping. So, I’ve tried to cut some of them off” (#10, woman, 18 years). Such accounts underscore the relational complexities participants navigated in their efforts to quit. Another participant captured the nuanced interplay between family, peers, and environment, explaining:

*It’s a mixed support because my family are supporting me to quit vaping, and when I’m with them, the urge to vape is reduced, but when I go out and around my peers, I have the urge to vape. So, it depends on my location and the people around me.*

[#9, woman, 21 years]

Beyond social normalization, vaping also emerged as a coping mechanism for many youth and young adults, particularly as a way to manage stress or emotional challenges. Participants described how stress from work, school, or personal relationships often drove them to vape, framing it as an accessible and socially acceptable method of self-soothing. One participant shared, “The moment I feel stressed, the only thing that comes to my mind is to go and vape” (#9, woman, 21 years), while another echoed this, stating, “Most times, when I’m sad, when I’m frustrated, the only thing that helps keep me calm is when I vape” (#8, woman, 21 years). This dual role of vaping as both a normalized social behavior and a tool for coping further entrenched its presence in participants’ lives, making quitting especially challenging without alternative coping strategies.

Online platforms also played a dual role in participants’ vaping experiences. Social media, particularly TikTok and Reddit, served as both a source of inspiration and a site of conflicting messages. For some, seeing influencers share their quitting journeys provided motivation: “There’s a big wave on TikTok of influencers quitting vaping [.] that’s helped me a lot” (#4, woman, 23 years). However, others noted that the glamorization of vaping on social media often undermined their efforts to quit.

#### Quitting Vaping Is a Personal Decision That Does Not Always Mean Success

Participants consistently emphasized the deeply personal nature of quitting vaping, highlighting how individual motivations, readiness, and self-efficacy shaped their cessation journeys. While external factors, such as social or familial pressures, sometimes influenced participants’ decisions to quit, these were often secondary to internal drivers like health concerns or personal goals. Illustrating how the physical effects of vaping served as a powerful motivator, one participant described that:

*My lung health in particular, I definitely have damaged lungs at this point. It’s very hard to breathe [...] I’m out of breath when I go up the stairs, or if I’m walking at a faster pace, I definitely notice now it’s harder to actually breathe and take deeper breaths than it was beforehand.*

[#6, woman, 21 years]

Others framed their attempts to quit as milestones in personal growth, with one describing it as a personal goal: “I wanted to quit since the beginning of the year […] I saw it as a personal goal type of thing” (#5, man, 22 years).

Despite recognizing the importance of quitting, many participants acknowledged their success hinged on their confidence and readiness to commit to the process. One participant reflected, “It’s really hard because it always comes down to me and me making up my mind” underscoring the centrality of self-efficacy in navigating cessation; they go on to add: “I feel like it has to be really my decision, and I’m not there yet” (#3, woman, 24 years), highlighting how personal readiness can act as both a catalyst and a barrier. External pressures, such as social disapproval or encouragement from family and peers, were recognized but rarely decisive. Participants stressed the importance of making their own decisions, emphasizing the individualized nature of quitting. One participant encapsulated this sentiment: “It has to be your decision. There is no one else who can do it for you” (#4, woman, 23 years).

Participants expressed a desire for recognition of small milestones in their vaping cessation journeys, emphasizing the importance of celebrating progress rather than focusing on setbacks. The pressure to succeed often felt overwhelming, particularly when relapses erased their “vape-free streak,” which some described as belittling their overall efforts. This highlights the need for supportive approaches that frame quitting as a nonlinear process, allowing room for growth and resilience. While intrinsic motivation played a key role in initiating quit attempts, participants expressed that external reinforcement would be helpful in sustaining their efforts over time. Encouragement from peers, digital tracking of progress, and structured incentives were seen as important in maintaining motivation, particularly during periods of heightened stress or vulnerability.

Participants frequently used redirection strategies to manage cravings and navigate the challenges of quitting vaping. These approaches involved substituting behaviors, avoiding triggers, and restructuring their routines to reduce opportunities for vaping. Redirection was often seen as a practical and immediate method of coping, especially during moments of heightened cravings or emotional stress. A commonly reported technique was behavioral substitution, where participants replaced vaping with alternative activities or products. Simple actions such as chewing gum, using mints, or staying physically active were described as effective distractions during craving episodes; “How I cope is either a coping mechanism, like nicotine gum, mints, or distracting myself in some way” (#12, woman, 24 years). These substitutions served as manageable, low-barrier interventions, providing participants with tangible alternatives during moments of temptation. However, while these strategies were useful for managing immediate cravings, participants noted that without external reinforcement, it was difficult to sustain momentum over time, reinforcing the need for structured, ongoing support.

Reliance on self-discipline and willpower alone was frequently associated with challenges and relapses. Participants who attempted unassisted cessation methods, such as quitting “cold turkey,” often found the process unsustainable. While some expressed a desire to take control of their behavior independently, they acknowledged the difficulty of doing so without external support or tools: “Cold turkey takes a lot of willpower [.] It’s not something you can just wake up one morning and do” (#1, man, 19 years). These perspectives exemplify the limitations of self-reliance, particularly when facing strong nicotine cravings or deeply ingrained habits. As such, many participants expressed a desire for digital interventions that not only supported self-directed quitting but also provided extrinsic motivators, such as virtual rewards, positive reinforcement, and social accountability, to sustain long-term cessation efforts.

#### Digital Support as a Bridge Between Individual and Social Needs

Participants highlighted the potential of digital tools to address the dual needs of individual autonomy and social connection in their vaping cessation journeys. Digital interventions, such as mobile apps, were seen as capable of personalizing the quitting process while offering supportive, nonjudgmental spaces for connection with others. Many participants appreciated the adaptability of these tools, emphasizing the importance of features that cater to their individual progress and goals. That said, most participants disclosed little experience engaging with currently available mobile-based vaping cessation apps. When questioned about why they did not use apps in their quit journeys or their general opinions on cessation apps, youth and young adults shared a lack of knowledge about existing apps, a lack of confidence in the effectiveness of vaping cessation apps, and a lack of sustained engagement with the apps they had tried. This lack of confidence stemmed in part from perceptions that existing tools were outdated, impersonal, or not tailored to the realities of current vaping products or youth and young adults’ experiences. One participant shared their previous experience with an app, stating:

*I think the technology wasn’t there for it. I just think it wasn’t as up to date as it should have been [...] it was more catered to, like older style vapes and less like the newer disposable kind.*

[#4, woman, 22 years]

Building confidence in AR-based interventions will require addressing these gaps by ensuring relevance, personalization, and innovation. Participants indicated that future tools must feel current and responsive in terms of technology and content and include features that reflect the modern vaping landscape, such as support for disposable vape cessation, real-time feedback, and interactive engagement. Additionally, establishing credibility through trusted partnerships, transparent design, and co-creation with youth and young adults may help foster greater trust and perceived efficacy. That said, several positive attributes of potential app-based interventions were discussed.

For participants who valued anonymity, digital platforms offered an intriguing means of connecting with peers facing similar struggles without fear of judgment. Features like anonymous forums for peer support within apps were especially appealing, as they allowed participants to feel less isolated in their journeys. One participant explained:

*[It] doesn’t have to be like something like super, super social, but, you know, just reading other people’s experiences, maybe talking about your experience, that kind of stuff. I think that’s important.*

[#5, man, 22 years]

Many youth and young adults emphasized the need for a balance between social connection and privacy; this blend of community and privacy was viewed as a critical strength of digital tools, particularly for youth and young adults navigating the stigma surrounding vaping. While traditional peer interactions in person could reinforce vaping behaviors, structured, judgment-free digital communities were seen as a way to foster motivation and reduce feelings of isolation. This blend of community and discretion was identified as a crucial strength of digital interventions, particularly for youth and young adults navigating the stigma surrounding vaping and the complexities of quitting within their social environments.

Participants were particularly interested in the potential of incentives to enhance their vaping cessation efforts, viewing rewards as motivating and affirming their progress. Many appreciated the idea of earning in-app currency that could be used for virtual rewards or gamified features, fostering a sense of achievement and making the quitting process more engaging. Tangible incentives, such as e-gift cards, were perceived as especially appealing, offering practical and immediate benefits that reinforced their commitment to staying vape-free. A participant highlighted this interest, stating:

*When I heard virtual rewards and incentives, I got so hyped up [...] one thing I think would be effective was, if you go a certain amount of days without vaping, your virtual reward could be an entry for an even bigger prize [...] I think that having contests and having, you know, things of that nature will be beneficial. I think that also providing gift cards and, you know, coupons and discounts and all of those things, they will definitely help.*

[#1, man, 19 years]

Participants emphasized that incentives could create a positive reinforcement loop, celebrating progress while encouraging continued effort. Youth and young adults were open to abstinence verification with methods like saliva cotinine testing, thus highlighting an interest in app-based contingency management (CM).

Digital tools were also recognized as a platform for reflection and growth. Features such as journaling, self-reflection prompts, and progress visualization to understand their behaviors better and celebrate milestones; “kind of reflect on, oh, I’ve made it this far, you know, like I could keep going and use that as a motivator” (#6, woman, 21 years). Several participants emphasized the need for content that is positively framed. App-based tools were ultimately viewed as an opportunity to bridge the gap between self-directed quitting and the need for external encouragement, creating a holistic support system.

#### AR as a Catalyst for Personalized Support

##### Overview

Participants endorsed AR as a unique and innovative tool for enhancing their vaping cessation journeys by offering support and distraction during moments of vulnerability. AR’s immersive and interactive nature has the potential to allow participants to engage with interventions in ways that feel dynamic, responsive, and motivating. Participants ranked proposed AR-based features through a Zoom whiteboard activity, emphasizing their preferences for tools that combine motivation, engagement, and practical support. The rankings reflected strong enthusiasm for gamification and virtual rewards as the most desirable features, highlighting the appeal of progress tracking and achievement-based incentives. This is followed by health effects visualization, interactive quitting support, and trigger identification and management, reflecting a desire for educational reinforcement and real-time coping mechanisms. These findings underscore AR’s potential to provide a holistic, engaging, and youth-centered approach to vaping cessation by making support accessible and immersive, particularly during the most vulnerable moments of the quitting process.

##### AR as a Conduit to Nonjudgmental Real-Time Feedback

The potential for AR to provide nonjudgmental, anonymous support resonated with participants. Interactive elements, such as artificial intelligence (AI)–powered avatars or real-time feedback, were viewed as desirable for those seeking guidance without fear of stigma.

*I might find myself in a situation where I have nobody to talk to about this, where I’m too embarrassed to talk about this, you know what I mean, like, and then if I have [...] AI or a real human who is qualified to talk about this, I do think that would be a helpful thing.*

[#7, woman, 22 years]

For some, AI-based interactions were preferable to human-led support, as they offered privacy, autonomy, and an escape from social judgment. Others, however, valued the “human-like” presence an AR avatar could simulate during moments of need, appreciating the blend of technological convenience with empathetic support. Not all participants trusted AI, with some expressing a preference for human-led interactions, such as those with counselors, due to the nuances, emotional depth, and authenticity of human conversations. To foster trust in future AR applications that incorporate AI, participants emphasized the importance of transparency in how AI functions, the ability to customize or opt into human support, and ensuring that AI interactions feel empathetic rather than robotic. Hybrid models, which allow users to choose between AI-driven guidance and human-led support, may offer the flexibility and emotional safety needed to meet diverse preferences, thereby increasing user trust and sustained engagement.

Participants emphasized the importance of tailored content and motivational messaging in maintaining engagement and supporting long-term cessation. Personalized, positively framed reminders and notifications were seen as essential tools for reinforcing motivation without inducing guilt or shame. Rather than punitive or negatively framed alerts, participants preferred encouraging messages that acknowledged progress and reinforced their reasons for quitting. One participant suggested:

*A feature that could give motivational affirmations for the day, like you don’t have to wait today, like some of motivation that comes up pops up every day on my app to keep me motivated not to go back to vaping or stuff like that.*

[#9, woman, 21 years]

Features such as customizable reminders, goal setting, milestone celebrations, and daily motivational messages were viewed as ways to sustain engagement while making the quitting process feel supportive rather than restrictive. AR’s ability to deliver real-time, interactive reinforcement, such as visual progress tracking or immersive encouragement from an AI avatar, positions it as an ideal tool for keeping users motivated and engaged in a way that feels personalized, empowering, and nonjudgmental. One participant underscored the importance of personalization in making the intervention feel meaningful:

*I think that personalization is definitely going to be the core of this app [...] I think that that should be kind of the leading principle of the app, I think you should have the right to choose.*

[#1, man, 19 years]

Another participant highlighted the importance of individualized goal setting, reinforcing the need for an intervention that adapts to the diverse experiences of users:

*Everybody has their own, like, goals, right? Everybody has their own little path that they’re on. And if, for me, not vaping for two weeks is a big goal, it might not be for somebody else who’s hasn’t been vaping for like, three years, you know what I mean?*

[#7, man, 22 years]

These findings emphasize that offering multiple support formats, ranging from AI-driven assistance to human-led interactions, is crucial to accommodating diverse user preferences. While some participants prioritized privacy and anonymity, others sought genuine human connection, reinforcing the need for flexibility and choice in digital cessation interventions. By fostering a personalized, stigma-free, and engaging support system, AR has the potential to transform vaping cessation into a more interactive, accessible, and user-driven experience.

##### AR as a Tool for Distraction and Motivation

Participants viewed AR’s gamification capabilities as a compelling way to support their cessation efforts, with the potential to both distract them from vaping and motivate them on their journey. AR was uniquely positioned to provide structured engagement during cravings, transforming vulnerable moments into opportunities for reinforcement. Interactive games or immersive environments could serve as a constructive alternative to vaping, helping participants navigate stress and temptation. One participant captured this sentiment by explaining how AR could serve as an immediate, accessible distraction: “If you feel a craving, you open the app, and you have somewhere to go and something to do” (#12, woman, 24 years); another shared a similar thought saying, “an app like this [would] come in handy in case of situations where I would be tempted to vape” (#9, woman, 21 years). These elements aligned closely with participants’ broader use of behavioral redirection strategies, offering a structured and engaging way to manage cravings.

Many participants described the need for interactive and rewarding elements that could transform quitting from a rigid, pressure-driven task into an engaging and reinforcing experience. One participant highlighted this point, sharing that “the gamification and like the rewards and that kind of stuff that can be something that would make me stay more [consistent]” (#5, man, 22 years). Gamification through features such as progress tracking, interactive challenges, and virtual rewards were particularly appealing for their ability to reinforce positive quitting behaviors while making the process feel more like a game than a struggle.

Participants envisioned gamification as a blend of competition, community, and engagement, drawing inspiration from popular mobile games. One participant described how AR-based features could create a social yet anonymous competitive element, likening it to games such as Hay Day or Farmville, where users can build progress over time and engage in friendly competition:

*I love that idea. It kind of like, in my mind, the way that I like think about it is, like AR Hayday or Farmville [...] there’s like, a social aspect to that where, like, you compete with people and you grow your farm or whatever [...] you can kind of, like, make it a competition with your friends or whatever, and grow a community that way, but also have that anonymous, like, just that random person across Canada that you could just battle it out with for most days that you’re not vaping, which I think is really cool.*

[#4, woman, 23 years]

By integrating leaderboards, virtual rewards, and community-driven challenges, AR could provide both an emotional incentive and a distraction during cravings, reinforcing progress in an engaging and interactive way. However, participants emphasized that gamified elements should be designed thoughtfully and sensitively to avoid discouragement. One key concern was handling setbacks, as overly punitive design choices, such as resetting progress entirely after a relapse, could undermine confidence and motivation. One participant stressed the importance of maintaining momentum even after a lapse:

*If I do all of a sudden vape, I’m not going to obviously continue earning points, but I don’t lose all of them, or, like, I just don’t lose my progress, is what I’m saying. I just don’t want to lose all progress just because I made a mistake and just because I did what I did.*

[#7, man, 22 years]

These findings highlight the delicate balance needed in designing gamified interventions for vaping cessation. AR-based tools must provide engagement, distraction, and reinforcement without introducing discouraging setbacks that might deter continued efforts. A well-designed system that celebrates progress, fosters competition and community, and offers flexible, forgiving incentives has the potential to make quitting feel like an achievable and rewarding challenge rather than an isolating struggle.

##### AR Meets the Desire for Education and Real-Time Coping Mechanisms

Another valued feature of AR was its potential to provide health visualizations, offering users a dynamic way to track the benefits of quitting. Unlike static educational content, AR has the ability to immerse users in a personalized, real-time experience, reinforcing tangible progress in a visually engaging manner. Participants expressed interest in seeing how their lungs and body could heal over time, believing this could serve as a motivational tool to sustain their quit attempts. One participant emphasized the power of real-time feedback, explaining, “I think having that visualized in AR would be also beneficial, so that people could kind of see their progress on their own body in kind of real time as it were” (#12, woman, 24 years). Another participant appreciated the positive framing of such a feature, remarking, “I like the idea of it, like showing the effects of quitting, because then it’s not just like, Oh, you’re doomed, you know?” (#5, man, 22 years). However, some participants noted the need for balance, cautioning against overly graphic or negative imagery that could provoke fear or denial. This highlights the importance of creating visualizations that are both impactful and user-sensitive. Participants also indicated that an AR visualization feature on its own may not be enough to sustain user engagement, but it would be a useful educational feature when integrated into a more holistic app.

CET was introduced as a potential feature to help desensitize users to vaping triggers, but participants expressed strong skepticism about its effectiveness in the context of vaping cessation. Many feared that deliberately exposing themselves to cues such as virtual vaping devices or smoking scenarios could be counterproductive, intensifying cravings rather than reducing them. One participant described their discomfort with being reminded of vaping, stating:

*I can’t even look at a vape because it just immediately, like, reminds me of it, and then I start thinking about it [...]. I just start, like, hyper fixating on it [...] it makes the cravings worse [...] I personally wouldn’t open that feature and like try to see a cue, just because I know that it would just make me think about it, and it would just make everything worse.*

[#6, woman, 21 years]

Another participant echoed this sentiment, saying:

*[It’s] going to make me more agitated for vaping somehow [...] even seeing vapes on phone is just going to, like trigger me more to go to what I’m trying to quit.*

[#11, man, 21 years]

This skepticism reflects a broader hesitation around strategies that may feel emotionally taxing without clear evidence of their effectiveness in the context of vaping cessation. Despite concerns around CET, participants expressed strong interest in educational tools that help them identify personal triggers and develop actionable strategies for managing them. Rather than confronting triggers head-on, participants preferred interventions that provided personalized coping mechanisms. One participant shared the importance of practical alternatives, sharing a desire for learning:

*Different actual things you can do in the moment [...] techniques [like] you can just grab a water bottle with a straw or some like something like that.*

[#4, woman, 22 years]

While another participant indicated a desire to learn more about their personal triggers, “something that helps me is to become aware of, like, I guess, my triggers” (#5, man, 22 years). Participants valued the idea of combining personalized insights with practical suggestions for overcoming challenges, which could enhance their understanding of their vaping behaviors and empower them to develop healthier coping mechanisms.

These findings suggest that AR could play a critical role in trigger management, not just through direct cue exposure but by integrating real-time insights and behavioral guidance. For instance, AR could help users log their triggers, recognize patterns over time, and receive tailored coping suggestions in response to their cravings. By combining personalized insights with practical, interactive solutions, AR has the potential to empower users to understand their vaping behaviors, build self-efficacy, and develop healthier coping mechanisms that support long-term cessation success.

## Discussion

### Primary Findings

This study explored the perspectives of youth and young adults on vaping cessation, emphasizing the potential role of digital interventions, including AR, in supporting their cessation journeys. Using ID, the findings highlight the multifaceted influences shaping cessation behaviors, including technological, relational, and individual factors. Four themes were co-constructed: (1) social and cultural context play a role in youth and young adults’ experiences of cessation, (2) quitting vaping is an individual endeavor that does not always mean success, (3) digital support as a bridge between individual and social needs, and (4) AR as a catalyst for personalized support. These findings underscore the complexities of vaping cessation and the opportunities for innovative, youth and young adult–centered interventions that align with young people’s digital habits, motivations, and support needs.

Stepping back from the individual themes, this study makes a broader contribution to the literature by proactively ensuring that youth and young adults’ needs and preferences are embedded into intervention designs from the outset. Many existing vaping cessation interventions are developed with limited engagement from young people [28], potentially restricting uptake and effectiveness. By using ID, a methodology that prioritizes applied health research, this study was able to generate practical, actionable recommendations that align with youth and young adults’ lived experiences, digital habits, and cessation challenges. Theoretical frameworks for user-centered design, such as the Information Systems Research framework [37] or the BUS Framework, which incorporates principles from Behavior change theories, user-centered design, and Social marketing [38], can guide the integration of youth and young adults’ perspectives into digital health design, thereby ensuring that future interventions are not only evidence-based but also user-centered, increasing their potential for engagement, relevance, and long-term success.

The first theme emphasized the pervasiveness and normalization of vaping within youth and young adults’ social environments, reinforcing both initiation and continued use. Many participants started vaping in social settings, where it was framed as an expected part of social interactions, making cessation particularly challenging. The normalization of vaping was not only a driver of use but also a barrier to quitting, as participants reported experiencing peer influence, social expectations, and fear of social exclusion if they stopped vaping. These findings align with prior research showing that peer reinforcement plays a critical role in youth and young adults’ vaping behaviors [3-7]. Similar to Rahman et al [28], many participants attempted to distance themselves from social settings where vaping was prevalent, but this often resulted in social disconnection, reinforcing the need for cessation interventions that provide alternative forms of social support without requiring them to disengage from their social lives entirely.

Quitting vaping was described as a deeply personal and nonlinear process driven primarily by intrinsic motivations such as improving health, achieving personal milestones, or addressing financial concerns. This aligns with Self-Determination Theory [39], which highlights the role of autonomy and intrinsic motivation in sustained behavior change. While external pressures, such as encouragement from family or disapproval from peers, played a role in some participants’ decisions to quit, self-efficacy and personal readiness emerged as the most critical factors for success in theme two. Many participants described the push-pull nature of cessation, where they oscillated between the desire to quit and difficulties maintaining abstinence, often cycling between quit attempts and relapse. This reinforces the need for cessation interventions that are flexible and adaptable, allowing users to engage at different stages of readiness without being penalized for setbacks. While intrinsic motivation initiated the quitting process, participants emphasized that sustaining long-term abstinence required external reinforcement, such as celebrating small wins and receiving positive social support. These findings suggest that effective cessation tools should cultivate self-determined quitting efforts and integrate structured external reinforcements, such as peer accountability, personalized encouragement, and digital incentives, to help individuals remain engaged and resilient throughout their quit journey.

Importantly, the findings also point to the limitations of hard deadlines or rigid abstinence expectations for this population. Participants described traditional cessation models as often misaligned with their lived experiences, this may be particularly pronounced given the mental health challenges many youth and young adults face. This aligns with recent research analyzing Reddit posts about quitting vaping, where young adults emphasized the importance of self-love, self-grace, and a harm reduction mindset over perfectionism [17]. Rather than promoting immediate abstinence, participants preferred compassionate, flexible approaches that framed quitting as a gradual process. Digital cessation tools that offer positive reinforcement, celebrate small steps, and normalize nonlinear progress may be especially effective for supporting young people navigating emotional, social, and behavioral complexities in their quit journeys.

Aligned with both themes 2 and 3, participants expressed a desire for progress tracking and milestone recognition, reinforcing research on positive reinforcement and gain-framed messaging in behavior change interventions [16,27,40]. These findings underscore the importance of integrating and balancing intrinsic and extrinsic motivation in digital cessation tools, ensuring that interventions celebrate progress rather than focusing solely on quitting as an all-or-nothing goal. Intrinsically motivated participants found personalized goal setting, self-reflection tools, and progress visualization to be meaningful motivators, reinforcing their sense of autonomy and self-efficacy. Others were more extrinsically motivated, responding positively to the potential for incentives, social reinforcement, and gamification elements, highlighting the importance of offering diverse engagement strategies within cessation interventions. By integrating multiple forms of motivation, digital cessation tools can increase engagement, enhance long-term commitment, and better support the diverse needs of youth and young adults navigating their quit journey.

Findings from the third theme suggest that digital tools are viewed as a potential bridge between individual autonomy and social connection, offering customizable, nonjudgmental, and interactive support. Participants reported limited engagement with existing vaping cessation apps, citing a lack of awareness, skepticism about effectiveness, and difficulties maintaining engagement. This aligns with prior research indicating that many available app-based vaping cessation tools lack user-centered design and may fail to address many of the essential components of cessation, such as coping strategies for withdrawal or relapse prevention, reducing their long-term efficacy [41,42]. Despite this, participants still viewed digital platforms as promising tools for personalized support, including peer-driven support networks, where they could connect with others anonymously and without stigma. This aligns with Huma et al [27] and Rahman et al [28], who emphasized the importance of socially connected, nonjudgmental spaces in digital cessation interventions.

Participants were divided in their preferences for AI-driven versus human-led support, highlighting the need for flexibility in interventions. Some valued AI-driven interactions, such as chatbots or virtual avatars, are appreciated for their immediacy, privacy, and nonjudgmental nature, making them useful for on-demand support during cravings. Others preferred human-led interactions, emphasizing the emotional depth and relatability of counselors or peer mentors. Underlying themes 3 and 4, many participants emphasized privacy as paramount to ensure users feel safe and supported while engaging with these tools. As such, transparency in data collection and usage practices would be essential to building trust and encouraging sustained engagement among youth and young adults.

The final theme positioned AR as a uniquely valuable tool for vaping cessation; unlike traditional cessation approaches, which often rely on rigid goal setting and self-discipline, AR can facilitate adaptive, personalized support, integrating features such as gamification, behavioral tracking, and AI-powered coaching by superimposing digital images and objects into the user’s real environment. AR can also provide distraction, a key facilitator to quitting identified by Al-Hamdani et al [43], Pbert et al [16], Rahman et al [28], and Struik and Yang [17], offering youth and young adults somewhere to go during times of heightened cravings and stress. Participants expressed enthusiasm for gamified features, such as progress tracking, virtual rewards, and interactive challenges, which have been highlighted as ways to improve user experience and enhance engagement with digital health interventions [44-48].

The findings align with existing cessation literature, which identifies goal setting, self-monitoring, and social support as common behavior change techniques (BCTs) for smoking cessation success [49,50]. BCTs refer to an intervention’s specific, active components that aim to influence behavior by targeting psychological or contextual mechanisms of change [51,52]. McKay et al [41] highlight the need for vaping cessation apps to use specific BCT-based features known to support quitting. The strong emphasis participants placed on personalized goal setting and progress tracking in this study reflects BCTs such as “goal setting (behavior)” and “self-monitoring of behavior,” both of which have been implemented in successful smoking cessation interventions and have been shown to enhance user engagement and quit outcomes [49,50]. Similarly, the desire for positive social reinforcement and community-based encouragement resonates with BCTs categorized under “social support,” which were not only present across all of the Canadian smoking cessation websites analyzed by Struik et al [53] but were also associated with greater user satisfaction and perseverance through quit attempts.

Participants expressed an interest in incentive-CM, where verified abstinence is rewarded with tangible or virtual incentives. CM has demonstrated preliminary efficacy in vaping cessation [54,55], with Raiff et al [55] finding that all 8 participants in their trial achieved full vaping abstinence over 2 weeks using a CM-based intervention. CM is often limited by its resource-intensive requirements; however, using nonfinancial incentives, such as in-app rewards, badges, progress milestones, or vouchers, could make it more scalable and accessible. 55 Bridging this concept with theme 4 and AR’s immersive and interactive nature, integrating CM within an AR-based vaping cessation app could enhance these effects even further by making rewards feel more tangible and tied to user behavior [22]. For example, virtual rewards could be visually displayed within an AR environment, allowing users to physically interact with their progress or “level up” within a gamified cessation journey. The ability to merge behavioral reinforcement with real-time, immersive feedback in AR could create a stronger sense of achievement and motivation, potentially leading to greater adherence, long-term success, and increased sustainability compared to traditional CM approaches.

Participants highlighted the importance of education and real-time coping strategies, viewing AR as a useful tool for health visualization, as discussed in the fourth theme. Many expressed an interest in tracking their lung recovery over time, seeing improvements as a motivational reinforcement for staying vape-free. However, participants cautioned against overly graphic or fear-based messaging, favoring positively framed reinforcement. When AR-CET was introduced as a potential feature, participants expressed strong hesitancy, fearing that intentional exposure to vaping cues would intensify cravings rather than reduce them. CET may still be a valuble tool for vaping cessation, however further exploration is needed and should be approached with sensitivity. Instead, participants preferred AR-based tools that help identify personal triggers and provide real-time coping strategies, reinforcing the need for interventions that offer behavioral guidance alongside educational components.

Overall, this study is the first to explore youth and young adults’ perspectives on AR for vaping cessation, offering valuable insights into the challenges, barriers, and opportunities this population faces. The findings suggest that AR-based tools have the potential to enhance vaping cessation interventions by offering a dynamic, interactive, and user-driven experience. Beyond personalization and engagement, resilience-building and long-term support must be central to intervention design. Regular check-ins through apps or chatbots can provide ongoing support, ensuring users remain connected to their cessation goals even after initial success. By aligning with youth and young adults’ digital behaviors and preferences, AR-based tools can bridge the gap between self-directed quitting and structured support, ultimately making vaping cessation more engaging, accessible, and sustainable. Unlike traditional cessation programs, which often emphasize abstinence and will power, AR-based interventions can offer adaptive, personalized support, positioning them as an innovative and effective tool in the evolving landscape of vaping cessation among youth and young adults.

### Limitations and Future Research

This study offers valuable insights into youth and young adults’ perspectives on vaping cessation and the potential role of AR-based interventions; however, several limitations should be acknowledged. The sample size was appropriate for the study’s exploratory and qualitative nature, as ID prioritizes depth and applicability over broad generalizability [31,32]. While the findings provide valuable insights into youth and young adults’ vaping cessation experiences, they reflect a specific group of participants, so they may not encompass the full spectrum of cessation behaviors across diverse populations. The inclusion criteria encompassed youth and young adult participants aged 16‐24 years; however, only 2 participants were 19 years old or younger, thus limiting the study’s capacity to capture the experiences of younger youth. While diverse in gender and vaping experiences, the findings may not fully capture the perspectives of harder-to-reach populations, such as individuals who have never attempted cessation, those from lower socioeconomic backgrounds, or those with limited access to digital technologies. Future research should investigate the barriers to engagement with digital cessation tools among underrepresented groups to ensure that interventions are inclusive and accessible. The small, exploratory sample held limited information power, particularly regarding gender-based differences. As Donaldson et al [56] noted, gender and sexual minority groups are at higher risk for vaping but remain underrepresented in cessation research. By incorporating gender as a disaggregating construct, this study emphasized its importance in understanding vaping cessation. Future research using mixed methods approaches or larger samples could better capture gender-specific influences, ensuring interventions are inclusive and responsive to diverse needs. Including a wider range of demographics and vaping histories in future studies could further refine and expand the applicability of these findings, ensuring that intervention recommendations are responsive to the varied needs and contexts of those attempting to quit vaping.

Second, the study relied on self-reported experiences, which may be subject to recall bias or social desirability effects. While qualitative methods provide rich, in-depth insights, future studies could incorporate longitudinal designs to track how youth and young adults’ experiences and cessation strategies evolve over time, particularly in response to real-world engagement with AR-based interventions. Additionally, experimental or mixed methods approaches could complement qualitative findings by quantifying the effectiveness of specific AR features, such as gamification and AI-driven support.

Third, a key limitation of this study is the theoretical nature of AR discussions, which inherently limits the study’s alignment with ID. ID is intended to generate applied health knowledge that informs real-world practice; however, this study’s exploration of AR as a cessation tool was largely hypothetical, as participants did not interact with a tangible AR-based vaping intervention. Without direct engagement, participants’ perspectives were based on speculation rather than lived experience, limiting the extent to which findings can be directly translated into applied interventions. This limitation was mitigated by encouraging participants to interact with popular AR-based apps (eg, Pokémon Go) prior to the sessions to help foster a preliminary understanding of AR technology and its potential applications. Future research should prioritize pilot testing AR-based vaping cessation tools, allowing for direct user interaction and real-world evaluation of features such as gamification, behavioral tracking, and AI-driven support. AR interventions should be designed to foster both intrinsic and extrinsic motivation, leveraging personalized and adaptive mechanics to enhance user experience and adherence.

Conducting studies where participants actively engage with AR-based cessation apps would enhance the credibility and transferability of findings within an ID framework by grounding insights in actual user experience rather than hypothetical feedback. Further exploration of advanced features, such as AI-driven personalization coupled with AR, could enhance the adaptability of interventions by tailoring support based on users’ behaviors, progress, and engagement patterns. AI could provide immediate feedback and personalized cessation plans, reinforcing motivation and commitment while reducing the burden on health care professionals [57]. Additionally, given the increasing prevalence of dual substance use, future interventions should address the co-occurrence of vaping and cannabis use, recognizing that these behaviors often intersect and present unique cessation challenges [58]. Lastly, while this study focused on youth and young adults’ perspectives, future research should explore collaboration among end users, health care providers, digital health developers, and public health stakeholders to ensure that AR-based vaping cessation tools are evidence-based, youth-friendly, and effectively integrated into existing cessation frameworks.

### Conclusion

In conclusion, this study is the first to explore youth and young adults’ perspectives on AR for vaping cessation, offering new insights into how digital interventions can be designed to meet the evolving needs of young people. As vaping remains a pressing public health issue, particularly among youth and young adults, there is an urgent need for innovative, evidence-based interventions that move beyond traditional cessation approaches. By leveraging personalization, interactivity, and immersive technology, AR app-based tools have the potential to redefine vaping cessation support, making it more engaging, accessible, and effective. Building on this work, future research should focus on designing and pilot-testing AR interventions, including mixed methods work that evaluates real-world engagement and effectiveness. By combining co-designed elements, tailored features, and evidence-based strategies, digital interventions can empower youth and young adults to not only quit vaping but also sustain long-term behavior change and improve overall well-being.

## Supplementary material

10.2196/79674 Multimedia Appendix 1 Qualtrics screener and survey.

10.2196/79674 Multimedia Appendix 2 Semistructured interview guide.

10.2196/79674 Multimedia Appendix 3 Zoom-based whiteboard activity.

## Abbreviations

AI: artificial intelligence
AR: augmented reality
BCT: behavior change technique
CET: cue exposure therapy
CM: contingency management
ID: interpretive description
RTA: reflexive thematic analysis
