# Supplementary material for: Youth and Young Adults’ Perspectives on Augmented Reality–Driven Vaping Cessation Interventions: Interpretive Description Study
Source: JMIR XR Spat Comput. 2025 Dec 23;2:e79674. doi: 10.2196/79674 (PMC13202502; doi:10.2196/79674)
Supplement: Multimedia Appendix 1 [file xr-v2-e79674-s001.pdf]

Feature Awareness

Thank you for your interest in participating in our study!

Our goal is to learn more about what youth and young adults like/do not like in vaping cessation apps and how we can incorporate immersive technology, like augmented reality, into these apps to support better young people who vape in their quitting journey.

Participants who complete the full study will receive a $50 e-gift card (ex, Amazon, Lululemon, Apple, BestBuy, Starbucks) for their time!

Please answer the following questions to see if you qualify:

Are you between 16 to 24 years old?

- Yes
- No

Do you live in Canada?

- Yes
- No

Are you proficient in written and spoken English?

- Yes
- No

Do you have access to a stable high-speed internet connection?

- Yes
- No

Do you vape?

- ○ Yes
- ○ No

Are you motivated to quit vaping?

- ○ Yes
- ○ No

If you answered No to any of the above questions, we thank you for your time and interest in our project.

If you answered Yes to all of the questions, please continue forward to learn more!

Study Information

You have met the inclusion criteria for participation in our study!

Emerging research shows that many young Canadians who vape are now seeking support to quit vaping, and we want to help!

Who are we?
A group of researchers at the University of British Columbia Okanagan Campuses led by Dr. Laura Struik and Masters of Nursing student Karlee Fonteyne.

What are we doing?
Our study aims to collaborate with youth and young adults interested in quitting vaping to identify the strengths and limitations of existing vaping cessation smartphone applications and explore the potential for incorporating an augmented reality (AR) feature.

If you choose to be part of this study, you will be invited to participate in an online interview or focus group session at a time that best suits you. The online interviews will be held via UBC-licensed Zoom from servers in Canada, and the recordings will be done directly on the researcher's device. The interviews will take between 50-60 minutes. To give you an idea of the types of questions that will be asked, two examples are provided:

- *Have you used a vaping cessation app before, and what features did you like the most?*
- *Would this augmented reality feature help you in your cessation journey?*

You will also be asked to fill out a questionnaire with some demographic and general questions about your vaping experiences. You will receive a $50 e-gift card to thank you for contributing to this study. Vendors include Amazon, Starbucks, Tim Hortons,

PC Optimum, Walmart, Lululemon, and EB Games.

If you are still interested, please continue forward to learn more and sign the consent form!

Consent Form

Information & Consent

[Information & Consent]

Your consent statement means:
- **You understand the nature of this study.**
- **You consent to participate in this study.**
- **You consent to have your participation audio recorded.**

If you agree, please type your full name and sign.

Signature:

× SIGN HERE

clear

Please indicate the email you wish to be contacted at:

Demographics Survey

Almost done... this last part is to help us learn more about you and the trends in youth and young adult vaping. Please answer honestly! All of your responses will be kept confidential.

What gender do you identify as?

- ○ Male
- ○ Female
- ○ Non-binary / third gender
- ○ Prefer not to say
- ○ Other (please specify): ______

What age are you in years?

______

Which of the following best describes you? (select all that apply)

- ☐ Indigenous
- ☐ White/European
- ☐ Black
- ☐ Asian
- ☐ Hispanic
- ☐ Native Hawaiian or Pacific Islander
- ☐ Do not know
- ☐ Other (please specify): ______
- ☐ Prefer not to answer

Indigenous Identity (select all that apply):

- ☐ First Nations
- ☐ Metis
- ☐ Inuit
- ☐ Prefer to self-describe (please specify): ______
- ☐ Prefer not to answer

Are you a member of the LGBTQIA2S+ community?

- Yes
- No
- Prefer not to say

What province or territory do you reside in?

- British Columbia
- Alberta
- Saskatchwan
- Manitoba
- Ontario
- Quebec
- Newfoundland and Labrador
- New Brunswick
- Nova Scotia
- Prince Edward Island
- Yukon
- Northwest territories
- Nunavut

What is your highest level of education? (This includes what you may currently be enrolled in)

- Less than high school
- High school graduate
- Trade
- 2 year degree
- 4 year degree
- Professional degree
- Doctorate

The next two questions ask about belonging to these categories because our individual experiences may shape our thoughts and experiences about accessibility and support.

Do you identify with having any of the following (select all that apply):

- ☐ Anxiety
- ☐ Attention-Deficit/Hyperactivity Disorder (ADHD)
- ☐ Autism
- ☐ Eating disorder
- ☐ Mood disorder
- ☐ Obsessive-Compulsive Disorder (OCD)
- ☐ Post-Traumatic Stress Disorder (PTSD)
- ☐ Other (please specify): ______
- ☐ Prefer not to answer

How long have you been vaping?

- ○ Less than a month
- ○ Between 1-6 months
- ○ Between 6 months to a year
- ○ Greater than a year
- ○ Greater than 3 years

How often do you vape?

- ○ Several times per day
- ○ About once per day
- ○ A few times per week
- ○ Once per week or less

How many times have you tried to quit vaping?

- ○ I have not tried to quit before
- ○ 1-4 times
- ○ 5-10 times
- ○ More than 10 times

What is your primary motivation for quitting vaping?

- ○ Health concerns
- ○

- The financial costs of vaping
- Pressure to quit vaping from family, significant other, or friends
- Change in perception about vaping; it is ‘not cool’
- Tolerance break
- Other (please specify): ______

What strategies have you used to help quit vaping (select all that apply)?

- [ ] Gradual reduction
- [ ] Cold turkey
- [ ] Tolerance breaks
- [ ] Nicotine Replacement Therapy (NRT) (ex: nicotine patches, gum, lozenges, or inhalers)
- [ ] Pharmacotherapy (ex: bupropion (Zyban) or varenicline ((Chantix))
- [ ] Vaping cessation smartphone applications
- [ ] Text-messaging based intervention
- [ ] Social support groups (in-person or virtual)
- [ ] Professional counselling
- [ ] Alternative Therapies (ex: acupuncture, hypnotherapy, or mindfulness-based techniques)
- [ ] Other (please specify): ______

What vaping cessation app(s) did you/do you currently use? (Select all that apply)

- [ ] Kwit
- [ ] Quash
- [ ] Stop Vaping Challenge
- [ ] Crush the Crave - Vape Edition
- [ ] Quit Vaping
- [ ] Quit Vaping for Good
- [ ] Quit Vaping Addiction Calendar
- [ ] Aeris
- [ ] Escape the Vape
- [ ] Quit Genius
- [ ] No Vape
- [ ] Smoke Watchers
- [ ] Smokler
- [ ] Other (please specify): ______

Do you also use any of the following substances? (Select all that apply)

- ☐ Cannabis products
- ☐ Combustible tobacco cigarettes or cigars
- ☐ Chewing tobacco (ex: snus)
- ☐ Other nicotine-based products (ex: nicotine spray, nicotine pouches- Zyn, Velo, etc.)
- ☐ Recreational drugs
- ☐ Other (please specify): ________
- ☐ I do not use anything else
- ☐ Prefer not to answer

How long did you use/have you been using vaping cessation app(s)?

- ○ Less than 1 month
- ○ 1-3 months
- ○ 3-6 months
- ○ Over 6 months

Are you familiar with augmented reality (AR) technology?

- ○ I have no idea what this is
- ○ I know a little bit about AR and how it can be used
- ○ I have a firm grasp of immersive technologies, including AR

Block 4

Would you rather be a part of an online focus group session (2-5 people) or participate in an online individual interview with the researcher?

- ○ Focus group
- ○ Individual interview

Powered by Qualtrics
