# Supplementary material for: Youth and Young Adults’ Perspectives on Augmented Reality–Driven Vaping Cessation Interventions: Interpretive Description Study
Source: JMIR XR Spat Comput. 2025 Dec 23;2:e79674. doi: 10.2196/79674 (PMC13202502; doi:10.2196/79674)
Supplement: Multimedia Appendix 2 [file xr-v2-e79674-s002.docx]

**MULTIMEDIA APPENDIX 1**

**SEMI-STRUCTURED FOCUS GROUP GUIDE**

**Project Title: Youth and Young Adult-Driven Augmented Reality for Vaping Cessation**

**Purpose:** Invite young Canadians to share their experiences and preferences for designing a vaping cessation app incorporating an AR feature in a small focus group setting (~50-60 minutes).

| **Preparation (using Zoom for interviews)** |
| --- |

1. AV check
2. Recording ready
3. Materials for sharing uploaded
4. Facilitator notes
5. Ensure the Qualtrics survey has been complete by the participants before starting
6. Ensure consent has been received from the participants

| **Welcome/Instructions for Facilitators (5 minutes)** |
| --- |

Participants will be welcomed to the Zoom waiting room. Participants will be reminded of the purpose of the study and that participation is voluntary. Identity and permission to record will be confirmed. Participants will then be admitted to the main room. The code of conduct will be outlined (*be considerate, respectful, and collaborative when speaking and listening and mute when not speaking*). Participants will be asked if they have any questions before the recording begins and will be requested to have their cameras on. Introductions and a short icebreaker (*If you could take two things with you to a deserted island, what would you take?*) will be done before beginning the discussion and posing the questions.

| **Interview questions (~45 minutes)** |
| --- |

*I have some questions about your experiences using vaping cessation apps.*

| **Interview Focus** | **Questions:** |
| --- | --- |
| ***Rapport/***  ***Contextualization*** | 1. Have you tried to cut back or quit vaping?    1. What approaches have you tried? What has been more effective for you? 2. What has made quitting hard for you? 3. What has made quitting easier for you?    1. Have you felt supported in your cessation journey? |
| ***App Likes and Dislikes*** | 1. Have you tried any vaping cessation apps before?    1. What apps?    2. How long and frequently have you used the app?    3. What features on the app kept you coming back?    4. What features of the app did you dislike? |

*Now that we have discussed using a vaping cessation app, I have some questions about incorporating an augmented reality (AR) feature into a vaping cessation app.*

**Provide a brief explanation of AR:** *AR is a type of immersive technology that overlays digital interfaces upon the user’s real-time and real-life physical surroundings, allowing users to interact with digital content through devices like smartphones, tablets, or AR glasses while still being aware of their surroundings. It is seen in apps like Pokemon Go, Google Map, and Snapchat filters.*

| **Interview Focus** | **Questions:** |
| --- | --- |
| ***Augmented Reality Feature –*** *questions will be posed using the Zoom Whiteboard tool. Participants will be asked to rank suggestions 1 (best) – 5 (worst) with the option to add ‘stick note’ comments and suggestions* | I am going to describe some possible AR interventions that could be built out to be used on a mobile device.   1. **Health Effects Visualization**: Used as an educational tool, AR can simulate the short-term and long-term health effects of vaping on the user's body. For example, users can see visual representations of how vaping affects their lungs, cardiovascular system, brain, and overall health over time. Users could also see the health benefits of vaping cessation, i.e. how the body heals itself.    1. *Would you be interested in an AR feature that helps you visualize the health effects of vaping over time*    2. *How would you like this information to be presented? Realistic or animated?*   AR can be used to visually represent users' progress in quitting vaping by overlaying virtual charts, graphs, or timelines that show their achievements and milestones.   - 1. *Where do you currently look for vaping information?*  1. **Interactive Quitting Support**: AR can provide interactive support for users trying to quit vaping. For instance, users can access virtual support sessions where they interact with AI generated support in real-time, share their experiences and get encouragement to stay vape-free.    1. *Would you be interested in accessing virtual support sessions with an AR avatar in real-time to share your experiences and get encouragement?*    2. *Would you prefer the support to be human led or AI?*      1. **Virtual Rewards and Incentives**: AR can gamify the quitting process by offering rewards and incentives for reaching vaping cessation milestones. This could look like growing a virtual plant or pet with virtual currency collected from vaping abstinence or completing daily challenges. Users can unlock virtual badges, achievements, or rewards within the app, to purchase virtual goods or access premium features or the rewards could be tangible like coupons or discounts.    1. *Would you be interested in a feature that tracks your progress and evolves as you reach different milestones like a plant or pet?*    2. *Would you participate in AR-based challenges or competitions with others trying to quit?*    3. *What types of rewards or incentives would you like to see? Would a discounted gym membership be desirable?* 2. **Trigger Identification and Management (Cue Exposure Therapy)**: AR can help users identify and manage triggers. For example, users can use their smartphone camera to scan common vaping triggers, such as vape products, or shops or social settings where vaping occurs and receive virtual prompts or coping/stress management strategies to resist the urge to vape.   Through AR, users can also be exposed to trigger objects (e.g. vaping device) in a controlled virtual environment, allowing them to practice coping strategies and build resilience against cravings. By repeatedly exposing users to triggers in a safe and controlled manner, the app aims to desensitize them to these cues and weaken their association with the urge to vape.   - 1. *How would you feel about an AR feature that provides real-time feedback when you feel the urge to vape, such as showing you a calming scene or guiding you through a relaxation exercise?*   2. *Do you think features, such as meditation, cognitive behaviour therapy and relaxation would be helpful in an app specific to vaping cessation?* |
| ***Recommendations*** *– comments can be shared on Whiteboard or outloud* | 1. Which of these AR features is the most intriguing to you? Why?   *Go through features individually and discuss*   1. How important is it for you to personalize the AR features in the app, such as choosing different avatars, environments, or scenarios?    1. Do you consider goal setting an important part of your reduction or quit journey? 2. Would you like AR features that allow you to interact with friends or a support group within the app?    1. For example, could seeing some else’s progress in an AR challenge motivate you?    2. Would you prefer social interactions that are anonymous or connected to people you know? 3. What do you think the limitations would be with an AR based app feature? Do you have any concerns? 4. What would make you want to continue using an AR-based app for vaping cessation over time? 5. What might cause you to lose interest? 6. How frequently should reminders to use the app occur? 7. Are there any additional features that you would like within or external to the app to support you in quitting vaping? 8. If the app had a small cost associated with it would that deter you from downloading it? |

| **Closing: final comments (5 minutes)** |
| --- |

Thank you for joining me today and helping us understand more about the experiences of young Canadians using vaping cessation apps and how an AR feature could be incorporated. This information will help in the development of helpful cessation apps and help produce resources that support YYA like you with quitting vaping. We are at the end of this session, but do you have any final thoughts you would like to share before we leave?

**Stop recording**

**Extra details:**

- Ask about e-giftcard preference
- Ask if they want to seek published results
- Ask if they want to be contacted for future studies
