# Supplementary material for: Youth and Young Adults’ Perspectives on Augmented Reality–Driven Vaping Cessation Interventions: Interpretive Description Study
Source: JMIR XR Spat Comput. 2025 Dec 23;2:e79674. doi: 10.2196/79674 (PMC13202502; doi:10.2196/79674)
Supplement: Multimedia Appendix 3 [file xr-v2-e79674-s003.docx]

**Multimedia Appendix 3**

Zoom Whiteboard Activity Example


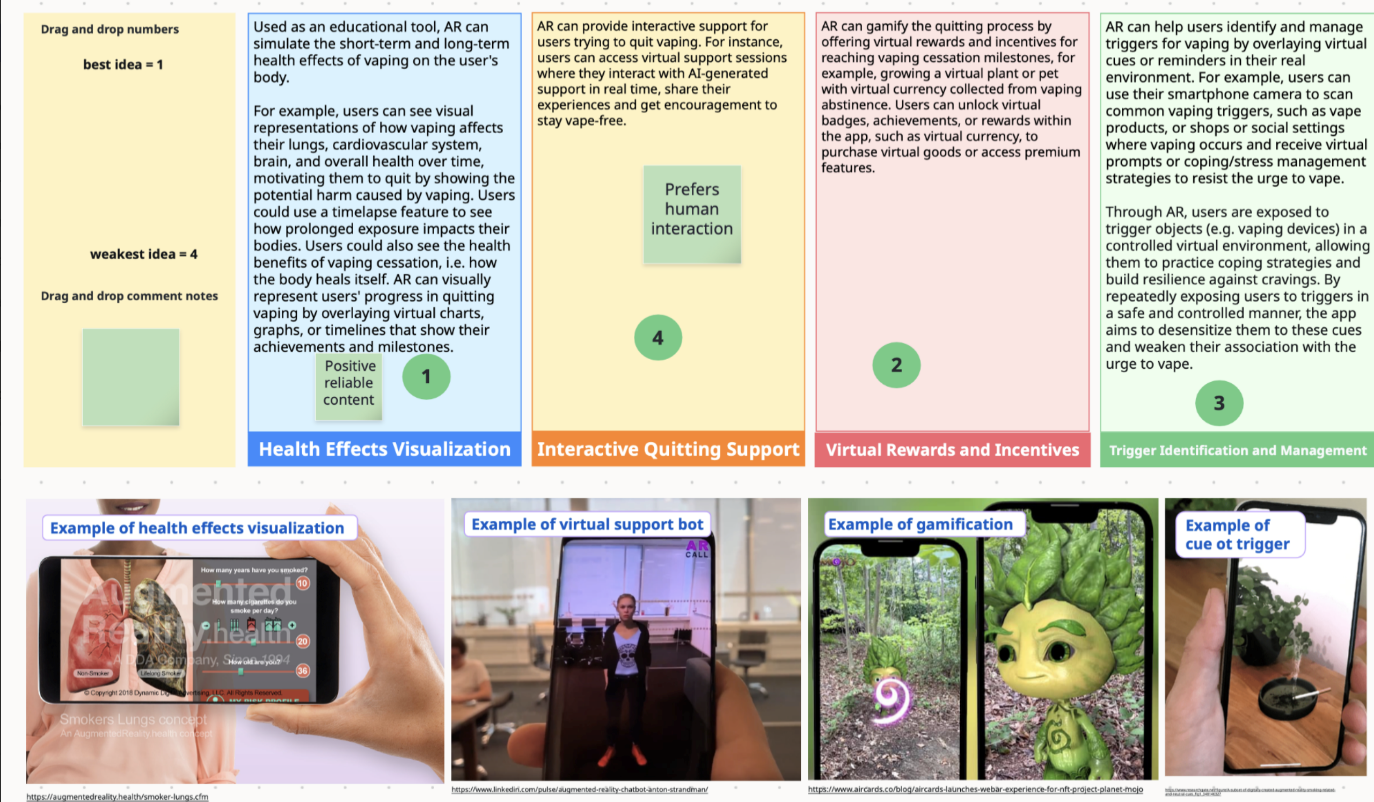


*Note*. Example of Zoom whiteboard activity showing an explanation of possible Augmented Reality (AR) feature examples with visual representation, and student ranking of the proposed AR features. Each student participated in this whiteboard activity during their Zoom interview session, and a screenshot was retained.
